# Clinical Factors Influencing Spontaneous Pleurodesis Success in Malignant Pleural Effusion in Patients With Indwelling Pleural Catheter

**DOI:** 10.7759/cureus.100854

**Published:** 2026-01-05

**Authors:** Hafiz G Kamil, Ahmad AbiMusaAsa'ari, Md Asaduzzaman, Jason Su, Mohammed Haris

**Affiliations:** 1 Respiratory Medicine, University Hospitals of North Midlands, Stoke-on-Trent, GBR; 2 School of Digital, Technology, Innovation and Business, University of Staffordshire, Stoke-on-Trent, GBR

**Keywords:** cancer patients, chronic renal failure, congestive cardiac failure (ccf), hypoalbuminaemia, indwelling pleural catheter, pleural effusion, pleurodesis

## Abstract

Background

Indwelling pleural catheters (IPCs) provide effective palliation for malignant pleural effusion (MPE) and may induce spontaneous pleurodesis (SP). However, risk factors causing transudative effusion, such as hypoalbuminemia, congestive cardiac failure (CCF), and renal failure (RF), may reduce pleurodesis success. Evidence on the impact of these comorbidities in MPE patients undergoing IPC placement remains limited.

Objective

To evaluate the impact of hypoalbuminemia, CCF and RF on successful SP in MPE.

Methods

A retrospective single-centre study was conducted in a tertiary care hospital on all patients with cytologically or radiologically confirmed MPE who underwent IPC placement between January 2020 and December 2024. SP was defined as catheter removal and no fluid recurrence up to 90 days. Demographic data, albumin levels, comorbidities, cancer type, and active anti-cancer therapy were compared between the SP and non-pleurodesis (NP) group.

Results

Among 110 patients (mean age 70 years; 54/110, 49% male), SP occurred in 30/110 (27%). Mean serum albumin was higher in the SP group (25.2 vs 20.4 g/L, p=0.001). CCF was present in 3/30 (10%) SP group compared with 15/80 (19%) NP group (p=0.005), while RF occurred only in the NP group, 4/80 (5%). Systemic anti-cancer therapy was associated with higher SP rates (26/30, 87% vs 32/80, 40%; p<0.001). SP varied by cancer type, highest in mesothelioma (6/12, 50%) and absent in small cell lung cancer (0/3, 0%).

Conclusions

Higher albumin, active anti-cancer therapy, and absence of multiple comorbidities causing transudative effusion predict successful SP. Identifying these factors may improve patient selection and procedural outcomes.

## Introduction

There are four factors that govern fluid movement to or from the pleural space: hydrostatic pressure, colloid osmotic pressure, filtration coefficient, and lymphatic function [1], which are described as Starling’s forces. When any of these factors are altered, fluid accumulates within the pleural space [1]. Malignant pleural effusion (MPE) is a common problem for people with cancer as a result of malignant infiltration of the pleura. It is usually associated with considerable breathlessness [2]. Installation of an IPC is usually an ambulatory procedure and involves the insertion of a tube attached to a one-way valve into the pleural space, allowing fluid to be drained from the pleural cavity as needed [3]. The indwelling pleural catheter (IPC) is an established therapeutic option recommended for the management of MPEs [4] and has been shown to provide symptom relief and induce spontaneous pleurodesis (SP) [5]. While SP may occur during the course of the disease, it is not universally achieved in all patients. SP rates have been reported up to 40% in MPEs [6].

Although previous studies evaluated IPC efficacy and chemical pleurodesis, few have examined clinical factors predicting SP success in MPE patients with IPCs. Understanding these predictors is essential to optimize management, avoid unnecessary procedures (i.e. chemical pleurodesis) in patients at risk of fluid reaccumulation, improve patient outcomes and prevent IPC-related complications.

Comorbidities such as hypoalbuminemia, congestive cardiac failure (CCF), and renal failure (RF) may potentially affect the likelihood of achieving SP. This is due to the complex interaction between the underlying malignant cause of MPE and systemic processes, such as coexisting transudative effusion, that can inhibit pleural adhesion.

This study evaluates the influence of hypoalbuminemia, CCF, and RF on SP success following IPC placement in MPE patients.

## Materials and methods

Study design and population

A retrospective cohort study was conducted at a single tertiary care National Health Service (NHS) centre in the United Kingdom, including all patients with cytologically or radiologically confirmed MPE who underwent IPC placement over a 5-year period (i.e., January 2020 to December 2024). A total of 110 consecutive patients were included.

IPC were drained at a standard of two to three times a week. SP was deemed successful when a catheter met the following criteria: (a) removal of the catheter without further effusion-directed intervention during the patient’s lifespan and (b) no evidence of effusion reaccumulation by clinical and radiographic evidence at 1-month post-removal [7]. In our study, the patient did not have any recurrence of fluid up to 90 days.

Inclusion criteria were cytologically or radiologically confirmed malignant pleural effusions. Exclusion criteria included prior talc pleurodesis, IPC complications post insertion (blockage, displacement, infection), or insufficient follow-up data.

Data collection

We extracted demographic data, albumin level (lowest serum albumin level during IPC placement period), presence of CCF (defined by pro-BNP >1500 or EF <35%), RF (estimated glomerular filtration rate (eGFR) <27 mL/min/1.73 m² with proteinuria), cancer type, receiving systemic anti-cancer therapy, and pleurodesis outcome.

Patients were followed until SP or death, with those dying prior to SP classified as non-pleurodesis (NP). SP was achieved in 26%. However, many patients die soon after catheter insertion, which precludes the chance of SP [7].

Statistical analysis

In our analysis, continuous variables were compared using a two-sample t-test. If the assumption of normality was not satisfied, we performed the Mann-Whitney U test (also known as the Wilcoxon rank-sum test). Categorical variables were analysed using the chi-square test for independence. A p-value of <0.05 was considered statistically significant. To show the strength of the relationship between variables, we also provided the effect sizes using Cohen’s d, or Cohen’s w measure. All statistical computations and data visualisations were performed in the R statistical computing environment, version 4.5.1.

## Results

Patient characteristics

A total of 110 consecutive patients with malignant pleural effusion underwent IPC placement during the study period. SP was achieved in 30/110 patients (27%), while 80/110 patients (73%) did not achieve pleurodesis (NP).

The mean age was 67.5 years in the SP group and 71 years in the NP group. Sex distribution was similar between groups, with 15/30 (50%) males in the SP group and 39/80 (49%) males in the NP group.

Albumin levels and concurrent pathologies

**Figure 1:**
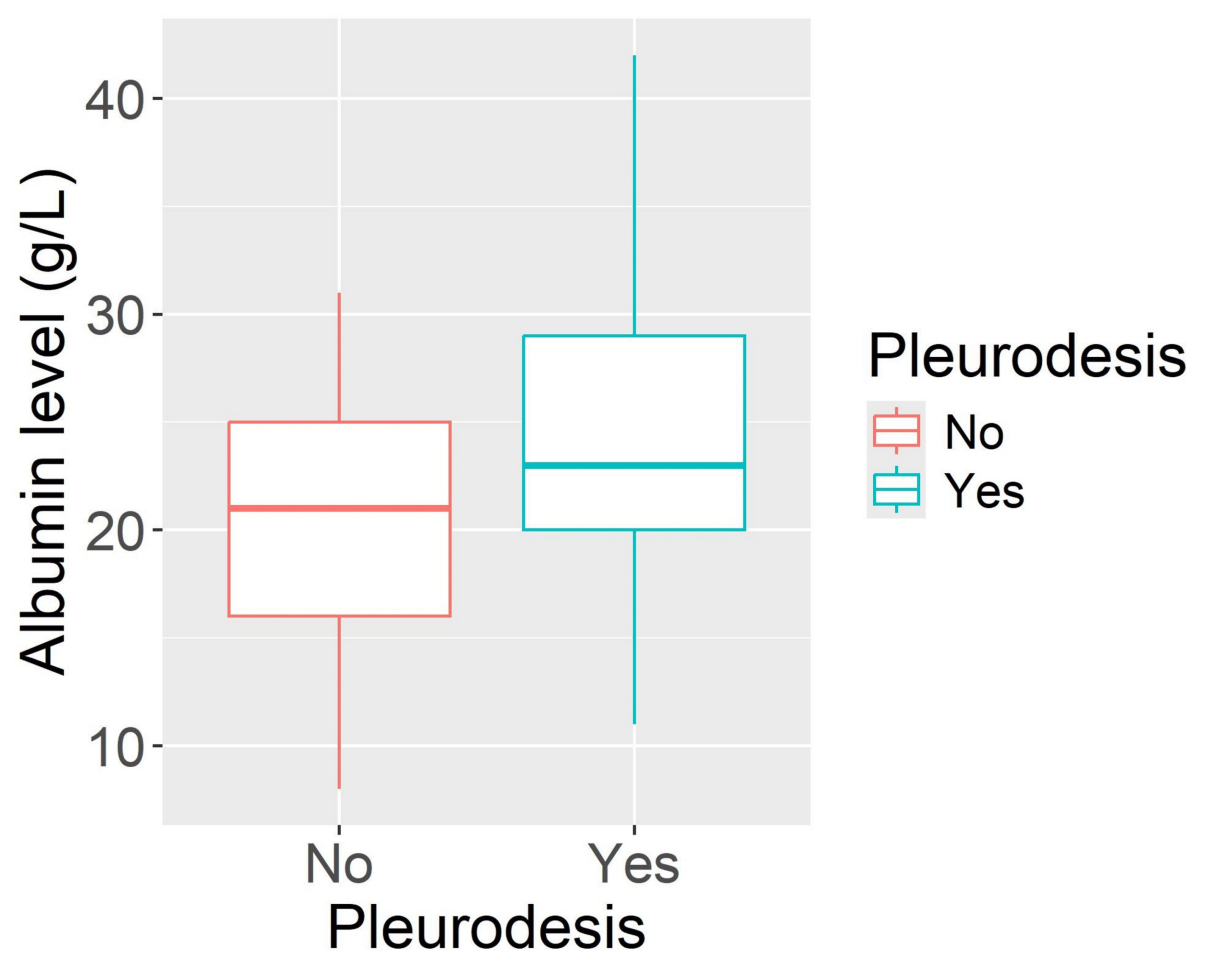
Box plot showing albumin level in spontaneous pleurodesis and non-pleurodesis groups.

Mean serum albumin was significantly higher in the SP group (25.2 ± 6.66 g/L, range 11-42) compared with the NP group (20.4 ± 5.26 g/L, range 8-31; p = 0.001) (Figure 1).

Hypoalbuminemia was present in 27/30 (90%) patients in the SP group and 77/80 (96%) patients in the NP group (p < 0.001).

CCF was observed in 3/30 (10%) patients who achieved SP compared with 15/80 (19%) patients in the NP group (p = 0.005). RF occurred only in NP group 4/80 (5%), but the sample size remained too small to calculate the p-value.

When the cumulative burden of risk factors causing transudative effusion was assessed, 3/30 (10%) SP patients had no risk factors, 24/30 (80%) had one risk factor, and 3/30 (10%) had two risk factors; none had all three. In contrast, among NP patients, none were without risk factors; 63/80 (79%) had one, 15/80 (19%) had two, and 2/80 (2%) had all three risk factors. A lower burden of the above comorbidities was significantly associated with SP (p = 0.032) (Table 1).

**Table 1:** Distribution of transudative pathologies between spontaneous pleurodesis and non-pleurodesis groups.

Condition	Spontaneous Pleurodesis (n=30)	Non-Pleurodesis (n=80)	Test: Effect Size, p-Value (Degree of Freedom)
Albumin level (g/L)	Range: 11-42 (Mean 25.2, SD 6.66)	Range: 8-31 (Mean 20.4, SD 5.26)	t-test: Eff. size = -0.84 p-val. = 0.001
Hypoalbuminemia	27	77	Chi-sq. test: Eff. size = 0.48 p-val. < 0.001 d.f. = 1
Congestive cardiac failure	3	15	Chi-sq. test: Eff. size = 0.67 p-val. = 0.005 d.f. = 1
Renal failure	0	4	---
No risk factors	3 (10%)	0	Chi-sq. test: Eff. size = 0.30 p-val. = 0.021 d.f. = 3
One risk factor	24 (80%)	63 (79%)	
Two risk factors	3 (10%)	15 (19%)	
Three risk factors	0	2 (2%)	

Anti-cancer treatment and spontaneous pleurodesis success

Receiving systemic anti-cancer therapy was significantly associated with successful SP. Among patients who achieved SP, 26/30 (87%) were receiving systemic anti-cancer therapy, compared with 32/80 (40%) in the NP group. Conversely, patients on best supportive care were more common in the NP group (48/80, 60%) than in the SP group (4/30, 13%). Overall, this association remains statistically significant (p < 0.001) (Table 2).

**Table 2:** Association between anti-cancer treatment and spontaneous pleurodesis.

Treatment	Spontaneous Pleurodesis (n=30)	Non-Pleurodesis (n=80)	p-Value
Best supportive care	4 (13%)	48 (60%)	Chi-sq. test: Eff. size = 0.40 p-val. < 0.001 d.f. = 1
Systemic Anti-Cancer Therapy	26 (87%)	32 (40%)	

Cancer type

SP rates varied according to the underlying malignancy. SP was most frequent in patients with mesothelioma (6/12, 50%), followed by non-small cell lung cancer (10/37, 27%) and extra-thoracic malignancies (14/58, 24%). No patients with small cell lung cancer achieved SP (Table 3).

**Table 3:** Association between cancer type and spontaneous pleurodesis.

Cancer Type	Spontaneous Pleurodesis	Non-Pleurodesis
Non-small cell lung cancer	10/37 (27%)	27/37 (73%)
Small-cell lung cancer	0/3 (0%)	3/3 (100%)
Mesothelioma	6/12(50%)	6/12 (50%)
Extra-thoracic cancers	14/58 (24%)	44/58 (76%)

## Discussion

Our study demonstrates that hypoalbuminemia, CCF, and RF are key factors negatively associated with SP success in MPE patients with IPCs. Statistical analysis confirmed that higher serum albumin levels were significantly associated with greater SP rates (p = 0.002). The poor outcome with hypoalbuminemia aligns with other studies; hypoalbuminemia was found to be associated with older age, lower Hb level, higher serum creatinine level and higher Charlson Comorbidity Index (CCI), as well as poor outcomes [8]. Hypoalbuminemia is possibly an independent predictor of all-cause mortality in patients with acute or chronic heart failure [9].

The presence of multiple risk factors causing transudative effusion was significantly associated with pleurodesis outcome (p = 0.032), indicating that an increasing comorbidity burden impairs the likelihood of SP. CCF was significantly more common in the NP group compared with the SP group (p = 0.005), while RF occurred exclusively in NP patients; however, the number of RF cases was too small to allow meaningful statistical analysis. Randomised controlled trial of the use of IPCs in patients with pleural effusions secondary to heart, liver or RF, found no difference in mean breathlessness scores, as assessed by daily visual analog scale (VAS), between the use of IPCs and as-required therapeutic drainage over a 12-week study period [10]. In our study, pleural effusion was exudative in nature, so it is consistent with other studies. In heart failure, pleural effusion meets the biochemical characteristics of a transudate, although in 25% of cases it may fall into the exudative range [11]. Pleural effusion in chronic kidney disease could be transudative or exudative [12].

Treatment status emerged as another strong predictor: patients receiving systemic anti-cancer therapy were significantly more likely to achieve SP (p = <0.001). This may reflect both disease control and the indirect benefits of improved physiological reserve. The lack of SP in small cell lung cancer patients and the relatively higher SP rate in mesothelioma further suggest that tumour biology influences outcome. Systemic anti-cancer therapy improves pleurodesis rates, possibly by controlling malignancy and mitigating comorbidities contributing to fluid accumulation. This is in line with Farooq et. al., chemoradiotherapy and immunotherapy post-IPC placement were associated with the development of SP among patients with MPE [13]. Additionally, a study to develop a predictive normogram for SP also showed systemic treatment was associated with successful SP [14]. Resolution of malignant pleural fluid was associated with a significant survival benefit, even when accounting for factors such as placement of an IPCs, anti-cancer therapy [15].

Collectively, these results suggest that albumin level, overall comorbidity burden, coexisting transudative pathologies and treatment status are crucial predictors of SP success in MPE patients with IPCs. Identifying high-risk patients’ pre-procedure could guide clinicians toward alternative strategies, such as early chemical pleurodesis, more aggressive comorbidity management, or prioritisation of systemic therapy before IPC removal attempts.

While this study identifies possible novel factors influencing SP within the context of MPEs, its conclusions are limited by the retrospective design, a modest sample size, early mortality and the absence of multivariate analysis. Further clarification of these clinical factors will require larger, prospective investigations to avoid selection bias.

## Conclusions

Higher serum albumin, absence of multiple risk factors causing transudative effusion, and ongoing systemic anti-cancer therapy are associated with potentially increased likelihood of SP in MPE patients with IPCs. Early identification of low-probability cases may guide targeted interventions and reduce unnecessary IPC retention.
